# Understanding GroEL and DnaK Stress Response Proteins as Antigens for Bacterial Diseases

**DOI:** 10.3390/vaccines8040773

**Published:** 2020-12-17

**Authors:** Kezia R. Fourie, Heather L. Wilson

**Affiliations:** 1 Department of Veterinary Microbiology, Western College of Veterinary Medicine, University of Saskatchewan, Saskatoon, SK S7N 5B4, Canada; kezia.fourie@usask.ca; 2 Vaccine and Infectious Disease Organization-International Vaccine Center (VIDO-InterVac), Saskatoon, SK S7N 5E3, Canada

**Keywords:** chaperone, heat shock protein, stress protein, DnaK, GroEL, adjuvant, bacteria, virulence factor

## Abstract

Bacteria do not simply express a constitutive panel of proteins but they instead undergo dynamic changes in their protein repertoire in response to changes in nutritional status and when exposed to different environments. These differentially expressed proteins may be suitable to use for vaccine antigens if they are virulence factors. Immediately upon entry into the host organism, bacteria are exposed to a different environment, which includes changes in temperature, osmotic pressure, pH, etc. Even when an organism has already penetrated the blood or lymphatics and it then enters another organ or a cell, it can respond to these new conditions by increasing the expression of virulence factors to aid in bacterial adherence, invasion, or immune evasion. Stress response proteins such as heat shock proteins and chaperones are some of the proteins that undergo changes in levels of expression and/or changes in cellular localization from the cytosol to the cell surface or the secretome, making them potential immunogens for vaccine development. Herein we highlight literature showing that intracellular chaperone proteins GroEL and DnaK, which were originally identified as playing a role in protein folding, are relocated to the cell surface or are secreted during invasion and therefore may be recognized by the host immune system as antigens. In addition, we highlight literature showcasing the immunomodulation effects these proteins can have on the immune system, also making them potential adjuvants or immunotherapeutics.

## 1. Introduction

Bacteria gain entry into a host through damage to the skin or contact with the mucosa whereupon they must invade or otherwise traverse the mucosal barrier to invade the host [1]. Exposure to these new environments can mean the bacteria experience changes in temperature, nutrient availability, osmolarity, and pH, all of which trigger a stress response and influence expression of heat shock proteins (HSPs) [2,3,4]. Induced proteins may include chaperones that assist in the folding of newly synthesized proteins, prevent aggregation of proteins during heat shock, and repair proteins that have been damaged or misfolded by heat shock, all without being part of the final protein structure [5]. These proteins are part of the constitutive proteome, but their expression is increased when the need arises. While the notion of heat shock proteins as immunogens or adjuvants may seem counterintuitive, they have been demonstrated to be potent vaccine components. Briefly, HSPs have been used in phase I and phase II clinical trials for a glioblastoma vaccine [6], a trial for a protective H9N2 influenza vaccine in chickens [7], and a phase II clinical trial for a type 1 diabetes therapy [8]. In addition, they stimulate the immune response when used in part as a therapeutic agent and they can elicit innate, humoral, and cell-mediated immunity. It is important to understand the traditional and non-traditional roles of these evolutionarily conserved proteins in order to fully evaluate their potential for uses in vaccines or as immunotherapeutics without giving rise to complications such as induction of autoimmunity. This review investigates how proteins whose functions were originally identified as HSP or chaperones have been re-evaluated to show that they play a role in bacterial virulence and that they may be suitable vaccine antigens, immunotherapeutics, or adjuvants.

## 2. Role of GroEL and DnaK in Bacterial Protein Folding

During synthesis of large proteins, chaperones delay chain compaction and prevent misfolding until proper protein tertiary structure can be achieved [9]. Chaperones work in concert with other chaperones to complete this task. For example, the bacterial ribosome-binding chaperone, Trigger Factor, interacts with the nascent polypeptide and Trigger Factor may be essential for recruitment of DnaK to the folding protein [10]. The details of chaperone pathways have been extensively reviewed elsewhere, therefore only a brief summary follows. DnaK, supported by the presence of the cofactor DnaJ, a HSP40 homolog, interacts with the nascent polypeptide [11,12]. DnaJ accelerates the hydrolysis of ATP bound to DnaK which is critically required for DnaK to bind to the polypeptide chain [13,14]. Release of the polypeptide chain is then aided by GrpE in the presence of adenosine triphosphate (ATP) [15]. Loss of DnaK may increase the dependence on chaperonin machineries to GroEL/GroES in some bacteria [16], indicating some level of communication between different chaperone/chaperonin pathways. Group 1 chaperonins such as GroEL/ES protect the folding protein from aberrant interactions or premature degradation in the cytosol [11,17,18]. Group 1 chaperonins differ from chaperones in that they protect the polypeptide chain from misfolding by providing a cage-like structure as protection from the environment [19]. ATP-bound GroES forms the “lid” for the GroEL cage. The polypeptide chain then folds inside the cage and is released after ATP hydrolysis or it may be recaptured for continual folding [20]. In *E. coli,* only 10% of the total proteome will utilize this GroEL/GroES pathway but while this may seem like a minor pathway, some of the proteins that rely on the GroEL/GroES pathway are essential, such as MetK and DAPA [21]. Specific assembly chaperones may assist the formation of oligomeric protein complexes by interacting with their folded subunits [22] or they may facilitate oligomeric assembly, transport to a selected subcellular compartment, and/or they help direct protein for disposal by degradation [6,23,24]. For instance, chaperones assist with the correct assembly and disassembly of nucleosomes to ensure that the distinction between transcriptionally active and transcriptionally repressed regions of the DNA is maintained [25]. Chaperones also assist in assembly of the proteasome that has a central role in removing abnormal or misassembled proteins, responses to stress, cell-cycle control, differentiation and metabolic pathway adjustments, and the cellular immune response [26]. A summary of traditional chaperone roles is included in Table 1.

## 3. Non-Chaperone Role of GroEL and DnaK in Prokaryotes

Chaperones may have roles in virulence and pathogenesis; this expansion of molecular roles can be defined as moonlighting—the ability of a protein to change roles in a yet undetermined manner [19]. Virulence factors help the bacteria to establish a system of infection through replication and persistence that may bring harm to the host, including promoting adhesion, invasion, evasion of host immune responses, and modification of host cell responses [36]. Understanding how GroEL and DnaK participate in these pathways to act as virulence factors may lead to novel targets for new vaccines or therapeutics.

GroEL (a HSP60 homologue) has been extensively explored as a moonlighting protein. GroEL from *Lactobacillus johnsonii*, *Mycoplasma pneumoniae,* and *Salmonella enterica* was discovered to relocate to the surface and may contribute in adhesion to mucin [28,29,30]. Mucins are a type of glycoprotein that make up the gel-like mucus found along the mucosal surfaces of the body that act as an obstacle to prevent colonization and invasion by pathogens [37]. For example, MUC1 and MUC4 are found in human milk and inhibit *S. enterica* infection [38]. Increased expression of MUC3 in the presence of *Lactobacillus spp* may inhibit disease caused by enteropathogenic organisms [39]. However, GroEL from *L. johnsonii* may play a role in mucin binding under the pH-specific conditions found in the colon [30,40]. In *S. enterica*, mucin binding has been proven to be critical to infection of intestinal epithelial cells [41]. Thus, some bacteria may have adapted by using GroEL to bind mucin as a way to get a foothold before initiating colonization or invasion. This role makes GroEL an attractive target for development of vaccines and therapeutics.

GroEL are secreted by some bacterial species including *Edwardsiella tarda*, *Helicobacter pylori*, and *Bacillus anthracis* [31,42,43]. Its functions while secreted are not known but it has been shown to bind iron in *H. pylori* which could possibly aid in competition with other bacterial species [31]. Iron binding is essential for *H. pylori* growth and obtaining free floating iron from the mucosal system would give it a competitive advantage [44]. It is not yet understood what causes GroEL to switch from being a protein synthesis supporter to being a virulence factor [45]. Its movement from the cytosol to the cell surface or the secretome without using canonical secretory systems is an underexplored avenue of research and the mechanisms leading to GroEL secretion have not yet been elucidated for any species.

An interesting secondary function of GroEL is the ability to act as a toxin in some insect species. Excreted GroEL of the species *Enterobacter aerogenes* paralyzed cockroaches but not mice [32]. Similarly, GroEL from *Xenorhabdus ehlersii* elicited an immune response in the larvae of *Galleria mellonella* that invaded the host *Steinernema longicaudum* [46]. Others showed that plants made to express GroEL from *Xenorhabdus nematophila* showed increased resistance to invading insects [47]. GroEL may exert species-specific effects as well as more general effects, making it not only a vaccine target, but also a novel therapeutic that requires further investigation.

DnaK (a HSP70 homologue) has also been implicated as a moonlighting protein. Surface-associated DnaK may be involved in adhesion interactions with eukaryotic plasminogen in *Mycoplasma pneumonia* [28], *Neisseria meningitidis* [33], and *Listeria monocytogenes* [34]. A study found that *Leptospira interrogans* was able to evade the immune system by binding first to plasminogen, then plasmin, leading to the degradation of essential complement proteins that helped *L. interrogans* survive [48]. Plasminogen is a glycoprotein located throughout the body that is degraded into its active form, plasmin [49,50]. While for many other species the necessity of binding to plasminogen has not yet been fully elucidated, the knowledge that DnaK can relocate to the surface and act as a binding protein should be investigated as an important avenue of vaccine research.

Loss of either GroEL or DnaK has pleiotropic negative effects on the cell either in regards to survival or loss of other cellular processes. When these genes were knocked-out in *Streptococcus intermedius*, several effects were observed. The DnaK-knockout bacteria became sensitive to increased temperature and were unable to grow at severe temperatures suggesting that DnaK was essential for surviving fever in a host. DnaK-knockout bacteria grew at a slower rate than wild-type strains [27] but pathogenicity was not affected, suggesting other pathways or factors affect the virulence of this bacteria. Similarly, a *S. aureus* DnaK-knockout mutant was also heat sensitive, grew slower, and the bacteria showed decreased pathogenicity relative to the wild-type bacteria [35]. The *S. aureus* DnaK-knockout mutant was also more susceptible to therapeutic agents in that the minimum inhibitory concentrations (MICs) did not change but the effect of the antibiotic on bacterial growth was more pronounced [35]. These results suggest that the chaperone DnaK is essential for survival and contributes to disease pathogenesis by playing a role in evasion of host immune responses. Interestingly, in the Gram-positive *Clostridium difficile* and Gram-negative *Escherichia coli* species, DnaK knockout mutants also showed a loss of *fliC* expression, a protein involved in flagellar synthesis that is a well-known immunogen [17,51,52]. These results suggest that expression of the *fliC* gene is under the control of DnaK or another downstream protein dependent on DnaK. While the *fliC* gene expression was reduced, *C. difficile* DnaK knockout mutants showed increased expression of the chaperonin *groEL* and its cofactor *groES* [17], which may suggest that the bacteria compensate for the loss of DnaK by increasing expression of other chaperones in an attempt to maintain proteomic homeostasis. A summary of non-traditional chaperone roles is included in Table 1.

## 4. Evidence for Immunogenicity of Prokaryote-Derived GroEL and DnaK

Proteins that are involved in bacterial pathogenesis are possible vaccine targets and establishing the immunogenicity of these proteins may be an important step in the development of an effective subunit vaccine. Here we discuss the different approaches used to establish the immunogenicity of GroEL and DnaK for the purposes of subunit vaccines.

It has been previously established that GroEL plays a role in *H. pylori* infection. Mice immunized with recombinant (r)GroEL showed induction of antibody-mediated immunity [53] and antibodies specific for *H. pylori* GroEL were reported in mothers and young children [54]. A research group used bioinformatics analysis to identify GroEL and other antigenic epitopes from *H. pylori* for use in a vaccine [55]. They identified five GroEL epitopes recognized by either MHC class I or class II that they included in the final vaccine composition. The final *H. pylori* antigen subunit construct was tested for Toll-like receptor (TLR) binding and was found to interact with TLRs 2, 4, 5, and 9 which suggests that the antigen itself could trigger innate immune responses [55]. While this is promising, vaccine safety, efficacy and protection of the expressed protein needs to be validated using in vivo experiments. By strictly using in silico approaches, one may miss epitopes or protein effects that can be identified by using in vivo approaches. A combination approach may be more encompassing.

Several vaccines have used GroEL as an immunogen. Mice vaccinated with the Bacillus Calmette-Guerin (BCG) vaccine responded with GroEL-specific CD8+ T cell immunity against *M. tuberculosis* [56]. Flounder immunized with a DNA vaccine coding for pCG-GroEL from *Edwardsiella tarda* showed activation of sIg+ lymphocytes, induction of memory T lymphocytes, and protective immunity against edwardsiellosis [42]. GroEL was also identified as an antigen for *Streptococcus agalactiae* [57] and *Lawsonia intracellularis* [58] through a combination of 2D electrophoresis and bioinformatics approaches. Briefly, 2D gel electrophoresis allowed for the separation of proteins from complex samples such as infected tissues [59]. Western blotting identified immunogenic bacterial proteins bound by antibodies from hyperimmune sera. However, this approach only identifies targets that promote induction of humoral immunity but not cell-mediated immunity. Alternate techniques could include a similar methodology such as the one described above where online resources identified epitopes for a vaccine that could potentially promote humoral and cell-mediated immunity [55]. GroEL orthologs were shown to be immunogenic in diseased hosts, including animals infected with *Helicobacter pylori* [60], *Ehrlichia risticii* (the causative agent of Potomac horse fever [61]), *Ehrlichia chaffeensis* (the causative agent of human monocytic ehrlichiosis [62]) and *Pasteurella haemolytica* (the causative agent of bovine pneumonic pasteurellosis [63]). Both DnaK and GroEL were identified as antigens against Nocardia species through mass spectrometry [64]. Interestingly, DnaK from *Streptococcus pneumonia* was found to be immunogenic and antibodies specific for it were cross reactive with DnaK from several other Gram-positive bacteria but not human homologues or DnaK from *Staphylococcus aureus* [65]. These results highlight the potential to develop vaccines that promote immunity against multiple pathogens by including an immunogen that is cross protective across species.

Finally, using bacterial proteins in subunit vaccines may impact the microbiome of the host species. For example, commensal *Lactobacillis johnsoni* GroEL is located on the surface [30] and it is secreted by *Helicobacter pylori* [31]. A vaccine using *H. pylori* GroEL may then inadvertently also target *L. johnsoni.* Care should be taken to evaluate the chance of any cross reactivity and the potential implications of it.

## 5. Evidence for Adjuvant and Immunomodulatory Potential of Prokaryote-Derived GroEL and DnaK

A protein that promotes an immune response in the absence of an antigen is an immunomodulator whereas that same protein formulated within a vaccine that can promote an antigen-specific immune response is referred to as an adjuvant. Immunomodulators and adjuvants are often classified according to their pattern-associated molecular patterns (PAMPs) that bind pattern recognition receptors (PRRs). For example, binding of a PAMP such as bacterial CpG and lipopolysaccharide (LPS) to TLR9 and TLR4, respectively, activates the innate system [66,67]. CpG is an immunomodulator and a vaccine adjuvant; unlike in eukaryotic cells, CpG islands in prokaryotes are unmethylated and are recognized as foreign by the eukaryotic immune system [68]. Methylation of the eukaryotic nucleic acids prevents cross reactivity with its TLR9 and therefore using CpG in vaccines does not promote autoimmunity [69]. Likewise, HSPs are one of the most evolutionary conserved proteins amongst all forms of life but prokaryotic HSPs promote immunity whereas the host HSPs are not generally recognized by its immune system, although there is some evidence for eukaryotic HSP involvement in some autoimmune diseases. The reason for this distinction is not clear and should be further explored. Prokaryotic and eukaryotic HSP60 (the class of HSP to which GroEL belongs) do share some common epitopes but also each have unique B cell epitopes so careful selection of epitopes may alleviate autoimmune induction concerns [70].

### 5.1. Immunomodulators

While chaperones such as HSPs are common to both mammalian and bacterial species, binding of bacterial HSPs to eukaryotic TLRs in cell lines has been well documented to induce innate immune responses. Thus it is also important to explore how GroEL and DnaK may act as innate immunostimulators and vaccine adjuvants. Endocytosis of chlamydial GroEL was found to signal through TLR2 and TLR4 and to activate stress-activated protein kinases c-Jun N-terminal kinase 1/2 (JNK1/2) and p38, the mitogen-activated protein kinase/extracellular signal-regulated kinase 1/2 (ERK1/2), and I-κB kinase (IKK) signaling pathways in murine macrophages [71]. Inhibition of clathrin-mediated endocytosis, which appears to be crucial for uptake and signaling of GroEL, blocked TLR4 signaling pathway [71]. Human and chlamydial HSP60 triggered activation of NF-κB complexes, leading to expression of inflammation-inducing cytokines [72]. Like GroEL, DnaK from *Streptococcus pneumoniae* was shown to induce TLR4 signaling in macrophages, leading to increased production of the inflammatory cytokines IL-6 and TNFα [73]. *Mycobacterium tuberculosis* HSP65 and HSP70 (the class of HSP to which DnaK belongs) were investigated for their effects on TLR signaling pathways in human dermal endothelial cells, macrophages, and HEK293 cells [74]. *M. tuberculosis* HSP65 and HSP70 signaled through TLR4 in human dermal endothelial cells to promote NF-κB activation whereas in macrophages, HSP65 signaled through TLR4 and HSP70 signaled through TLR2 and TLR4, the latter of which is dependent on the presence of MD2 glycoprotein [74]. DnaK added to an antibody treatment study in mice increased the antibody therapeutic potential but only when the antibodies were aggregated via heat and agitation [75,76]. Furthermore, delivery of *E. coli*-derived HSP70 to brain tumors was shown to induce production of Th-1 cytokines IFNγ and TNF-α and to decrease B cell populations in children, which potentially reduced tumor growth [77]. Thus, GroEL and DnaK may have potential as immunomodulators.

Interestingly, GroEL in mammalian cells has also been found to have non-canonical locations including the cell surface [78]. For example, it was found that T cells undergoing apoptosis express both HSP60 and HSP70 on their surface [79], stressed macrophages express HSP60 on their cell surface which is recognized by T cells specific for *M. tuberculosis* HSP60 [80], and stressed aortic cells have significantly increased surface expression of HSP60 relative to non-stressed cells [81]. While increased expression of HSPs during stress is expected, it not yet clear why eukaryotic cells relocate HSPs to their surface.

Care must be taken to ensure that the immunomodulators do not inadvertently exacerbate autoimmune diseases or impact disease severity. Arthritis is an autoimmune condition exacerbated by IFNγ as well as IL-17 production by Th17 cells [82,83,84,85]. Synovial cells from patients with arthritis that were incubated with *M. tuberculosis* HSP70 led to increased production of IL-10 [86]. Increased IL-10 production was correlated with decreased IFNγ cytokine production suggesting that these HSP70 may have potential to reduce disease severity in arthritis patients. In contrast, one study showed that human HSP70 increased the proportion of Th17 cells which exacerbated rheumatoid arthritis [87]. The safety of HSPs as immunotherapeutic agents will need to be investigated carefully lest they help against one disease state but exacerbate another.

### 5.2. Adjuvants

DnaK and GroEL can also modulate immune responses to antigens. By fusing a G-protein coupled receptor (GPCR) to *E. coli*-derived GroEL, mice showed significant induction of a humoral response and murine dendritic cells showed elevated production of IL-12p70 and IL-23 that was not present when mice were vaccinated with the GPCR alone [88]. Ovalbumin (OVA) peptides conjugated to DnaK or HSP70 increased MHC II processing in macrophages and dendritic cells relative to cells stimulated with peptide alone [89]. This signaling led to activation of CD4+ T cells independent of MyD88 or CD40. OVA conjugated to DnaK led to increased processing and increased peptide uptake but only in acidic environments, such as those found in vacuoles [89]. Antigen peptide conjugated to DnaK also showed enhanced peptide uptake and processing on MHC I which promoted CD8+ T cell activation [90]. Similarly, conjugating a malaria peptide to *Mycobacterium tuberculosis* HSPs 65 and 70 produced a strong humoral responses [91]. Together, these results suggest that GroEL and DnaK may be suitable as antigen carriers that lead to induction of cell-mediated immunity through both the CD4+ and CD8+ lineages, making them a diverse and enticing vaccine adjuvant. Likewise, the humoral and cell-mediated effects of a *Shigella* vaccine were enhanced when rGroEL was included as an adjuvant [92]. The ability to enhance both humoral and cell-mediated immune responses is an attractive feature as some commercial vaccines are primarily known for only eliciting one of the two arms of the immune system.

Similarly, the ability to have species-specific responses is another characteristic that separates HSPs from adjuvants that generally are recognized simply through PAMPs. In instances where targeting of a pathogen may also target a commensal organism, inclusion of a species-specific HSP in the vaccine may allow for specificity of immune activation or targeting. Being proteins, HSP can also be fused to antigens directly to induce immunity. For example, when developing a *Shigella* subunit vaccine, *Shigella* rGroEL was fused to the antigen IpaB as immunogenicity of the vaccine was enhanced when the two proteins were administered together [92,93]. The resulting fusion vaccine displayed a strong Th1/Th2 immune response while also providing high levels of protection in mice whereas the unfused antigens had a weaker response [93]. Not only does this demonstrate an advantage from an immunology perspective but also advantages from a manufacturing point of view. Fusion of antigen with adjuvant allows for increased efficiency and ease of production while also reducing the cost of production.

There is some concern that using the evolutionary conserved proteins DnaK or GroEL as adjuvants may potentially cause or aggravate autoimmune diseases. For example, the autoimmune disease Guillain-Barré syndrome has also been associated with infection by *Campylobacter jejuni* [93,94]. Immunoproteomic analysis showed that GroEL and DnaK were among the chaperones in *C. jejuni* that share high homology with human homologues that may contribute to Guillain-Barré syndrome triggering [95]. Recently, it was speculated that HSP70 may play a role in the autoimmune disease multiple sclerosis (MS) as genes related to HSP70 were overexpressed in MS patients [96]. A rather elegant study by Elfaitouri et al. demonstrated that there may be cross-reactivity between prokaryotic HSP60 and antibodies derived from patients with the autoimmune disease myalgic encephalomyelitis. The cross reactive antibodies derived from several prokaryotic species making it difficult to link a certain species with the disease [97]. It has also been suggested that HSP60 of *Yersinia enterocolitica* may play a role in autoimmune disease as increased antibodies to HSP60 were only found in patients who are predisposed to autoimmune diseases through the genetic marker HLA-B27 [98]. A survey of serum from patients with the autoimmune disease spondyloarthritis showed that patients had antibodies against bacteria-derived HSP60 but not human-derived HSP60 and there were no cross-reactivity between the antibodies [99]. Furthermore, the levels of antibodies against bacterial HSP60 but not human HSP60 were shown to be reduced in patients with inflammatory bowel disease [100]. The use of HSPs both as adjuvants and antigens seem promising and some data suggest that the chance of bacterial-induced autoimmune disease appears to be low. Clinical trials studying vaccines that contain HSPs as an adjuvant or antigen appear to be promising [7,8,9]. However, more studies should be undertaken to discern whether HSPs from prokaryotes in vaccines or as immunotherapeutics have a high degree of safety or can lead to an autoimmune disease.

## 6. Conclusions

Stress/heat shock proteins are a diverse group of proteins that are essential to the maintenance of bacterial and eukaryotic cellular function. Not only do they ensure that proteins are properly folded, but when under stress, bacteria express and relocate HSPs to aid in bacterial adherence, invasion, or immune evasion. This multitasking ability of HSPs reinforces the notion that one protein: one function is longer considered true. However, it is still not clear how these evolutionary conserved proteins can be so effective at acting as antigens, immunomodulators, and adjuvants, and they should be studied further. The discovery that HSPs can moonlight as novel vaccine candidates and adjuvants indicates that there may be more such adjuvants or immunomodulator candidates elsewhere in the prokaryotic proteome. Safety studies should be undertaken for each species-derived HSP intending to be administered in a vaccine or as therapeutic agents to ensure that the potential of inducing autoimmunity is negligible.

## Figures and Tables

**Table 1:** Traditional and non-traditional roles of DnaK and GroEL.

Chaperone/Chaperonin	Traditional Role	Source
**DnaK and GroEL**	Delay chain compaction	Balchin et al., 2020 [10]
	Assist in transport	Kim et al., 2013 [23]
	Direct proteins for degradation	Kim et al., 2013 [23]
	Assist oligomeric protein complexes	Ellis, 2006 [24]
	Assembly of proteasome	Baumiester et al., 1998 [26]
	Temperature homeostasis	Tomoyasu et al., 2012 [27]
**Chaperone/Chaperonin**	**Non-Traditional/Moonlighting Role**	**Source**
**GroEL**	Adhesion to plasminogen	Hagemann et al., 2017 [28]
	Adhesion to mucin	Ensgraber et al., 1992 [29]; Bergonzolli et al., 2006 [30]
	Iron binding	González-López et al., 2013 [31]
	Toxin	Yoshida et al., 2001 [32]
**DnaK**	FliC Expression	Jain et al., 2017 [17]
	Adhesion to plasminogen	Hagemann et al., 2017 [28] Knaust et al., 2007 [33] Schaumburg et al., 2004 [34]
	Bacterial growth	Tomoyasu et al., 2012 [27]
	Pathogenicity	Singh et al., 2007 [35]
	Therapeutic agent tolerance	Singh et al., 2007 [35]
